# Community Size as a Factor in Health Partnerships in Community Parks and Recreation, 2007

**DOI:** 10.5888/pcd10.120238

**Published:** 2013-07-25

**Authors:** Laura L. Payne, Jo An M. Zimmermann, Andrew J. Mowen, Elizabeth Orsega-Smith, Geoffrey C. Godbey

**Affiliations:** Author Affiliations: Jo An Zimmermann, Texas State University at San Marcos, San Marcos, Texas; Andrew J. Mowen, Geoffrey C. Godbey, Pennsylvania State University, University Park, Pennsylvania; Elizabeth Orsega-Smith, University of Delaware, Newark, Delaware.

## Abstract

**Introduction:**

Although partnerships between park and recreation agencies and health agencies are prevalent, little research has examined partnership characteristics and effectiveness among communities of different sizes. The objective of this study was to determine whether park and recreation leaders’ perceptions of partnership characteristics, effectiveness, and outcomes vary by community size.

**Methods:**

A web-based survey was completed in 2007 by 1,217 National Recreation and Park Association members. Community size was divided into 4 categories: very small, small, medium, and large. Questions measured agencies’ recognition of the need for partnerships, their level of experience, and the effectiveness and outcomes of partnerships.

**Results:**

Larger communities were significantly more likely to recognize the need for and have more experience with partnerships than smaller communities. Very small and large communities partnered significantly more often with senior services, nonprofit health promotion agencies, and public health agencies than did small and medium ones. Large and small communities were significantly more likely than very small and medium communities to agree that their decision making in partnerships is inclusive and that they have clearly defined goals and objectives. Large communities were significantly more likely than very small communities to report that their partnership helped leverage resources, make policy changes, meet their mission statement, and link to funding opportunities.

**Conclusion:**

Community size shapes partnership practices, effectiveness, and outcomes. Very small communities are disadvantaged in developing and managing health partnerships. Increasing education, training, and funding opportunities for small and rural park and recreation agencies may enable them to more effectively partner with organizations to address community health concerns.

## Introduction

Community park and recreation agencies are stakeholders in addressing health issues such as physical inactivity, obesity, and chronic disease and are partners in the healthy communities’ movement. Interorganizational partnerships can facilitate health behavior change that prevents chronic disease. Kernaghan (1) defined partnerships as relationships that involve a sharing of power, work, support, or information with others for the achievement of joint goals or mutual benefits. Partnerships are important for park and recreation agencies given health, social, and economic concerns of the nation (2,3). Bors and colleagues (4) emphasized the need for partnerships to address public health issues because various disciplines and groups can accomplish more together than separately. Most model health promotion programs (eg, Centers for Disease Control and Prevention’s Action Communities for Health, Innovation, and Environmental Change [ACHIEVE] Initiative [5]) require formal partnerships between health and park and recreation agencies to achieve their goals. Furthermore, in a study of North Carolina park and recreation directors, respondents indicated that one of their highest priorities was to develop more interagency health partnerships (6).

Health partnerships in parks and recreation have become more prevalent in the past 20 years as agencies try to optimize resources with limited budgets. Although the importance of health partnerships is widely recognized (4,6) little is known about the nature and extent of partnerships between park and recreation agencies and health agencies. One study found that health partnerships were common across the park and recreation field; approximately 90% of agencies report some type of partnership (7). Health partnership participation was found to be related to the size of the organizational budget and population of the community served. Agencies that served larger populations were more likely to engage in formal collaborations. Therefore, the purpose of this national study was to determine whether perceptions of partnership characteristics, effectiveness, and outcomes vary by community size.

## Methods

### Data collection

This study is part of a larger cross-sectional project that consisted of an online survey and in-depth interviews of park and recreation managers. Data were collected by using a web-based survey, and Pennsylvania State University’s institutional review board approved this study. The National Recreation and Park Association (NRPA) membership database was used to identify directors or senior managers (4,388 of 21,152 members met these criteria). Investigators reviewed the database to ensure that only 1 person from each member agency was invited to participate in the survey. NRPA is a professional organization whose mission is to advance parks, recreation, and environmental conservation efforts that enhance the quality of life for all people.

A presurvey letter was e-mailed to the sample on October 1, 2007, to announce the online survey. Ten days later, a second e-mail was sent with a cover letter and a link to the survey. After the first survey invitation, follow-up e-mails were sent to nonrespondents every 10 days for 1 month. Within the list of 4,338 NRPA members, 558 e-mails were undeliverable, reducing the sample to 3,780. Of these recipients, 1,217 completed the questionnaire, a response rate of 32%. Individuals contacted could opt out of the survey, take it and stop at any time, or complete the survey. Respondents concluded their participation when they finished the survey (which took 10–15 minutes) and were entered into a drawing for a small retail gift card. Because the focus of this study was local, public community park and recreation agencies (eg, municipal, township, county), state and national parks, military recreation, and nonprofit agencies (eg, YMCAs) were excluded from subsequent analysis, resulting in 1,089 usable surveys. To assess the potential threat of nonresponse bias in the sample, a random sample of 100 nonrespondents was contacted by telephone and e-mail and answered several questions. Results indicated that respondent characteristics and agency partnership practices reflected those reported by nonrespondents regarding agency type, community size, partnership participation, and effectiveness.

### Study measures

Community size consisted of 4 categories based on the population size served by the agency: 1) very small (< 30,000 residents), 2) small (30,000–60,000 residents), 3) medium (60,001–100,000 residents), and 4) large (≥100,001 residents). Partnership involvement was assessed by asking, “Within the past five years, has your agency/organization partnered with an outside organization (eg, health department, nonprofit, health care organization) to specifically promote health, wellness or physical activity within your community” (yes/no)? The number of partnerships was assessed by asking “With how many different health/wellness partnerships is your organization currently involved?” Two items developed by Hasnain-Wynia et al (8) were used to assess the degree to which their organization recognized the need for health partnerships and had experience with them on a 5-point scale (1, not at all; 3, somewhat; and 5, a great deal).

Partnership types were measured by a list of 13 partner types represented in the National Physical Activity Plan (9). Types were 1) public health, 2) nonprofit health promotion agencies, 3) hospitals, 4) planning organizations, 5) transportation agencies, 6) health insurance companies, 7) nursing home and assisted living centers, 8) colleges and universities, 9) schools, 10) local businesses, 11) senior services, 12) sports organizations, and 13) corporations.

Perceptions of partnership effectiveness were measured with 12 statements representing factors contributing to partnership effectiveness as established in prior partnership studies (8,10,11). Items assessed included decision making and conflict resolution, leadership and management, shared vision and trust, resource allocation, and communication. Examples of these statements are, “Our health partnership decision making is inclusive,” “Our health partnerships have clearly defined goals and objectives,” and “Stereotypes about partners from other professions have been broken.” They were rated on a 5-point Likert-type scale (1, strongly disagree; 2, disagree; 3, neither agree nor disagree; 4, agree; and 5, strongly agree). Content validity of these items was established in previous research, and authors recommended their use in future partnership studies (8,10,11). Finally, partnership outcomes were measured with the question “To what extent has your agency’s/organization’s involvement in health partnerships resulted in the following . . .”. A closed-ended list of outcomes followed and were answered on a 5-point scale (1, not at all; 3, somewhat; and 5, a great deal). The list was 1) leveraging additional resources (eg, money, personnel, equipment), 2) visibility, 3) image, 4) meeting the mission statement, 5) linking to funding opportunities, 6) changes in policy, and 7) improvements to physical features within the community. These items were developed in consultation with NRPA staff and by using themes that emerged from in-depth interviews with park and recreation managers and directors. Frequencies and descriptive statistics were used to describe the characteristics of the sample and their partnership engagement. Chi-square analysis was used to compare characteristics of partnerships by community size, and analysis of variance (ANOVA) was used to compare partnership effectiveness and outcomes by community size.

## Results

Local and municipal park and recreation agencies were most of the sample (892 [81.9%] organizations). There were also 96 (8.8%) county park systems and 101 (9.3%) special districts represented. Regarding community size, 409 (40%) agencies serve very small communities, 221 (21.6%) serve small communities, 139 (13.6%) serve medium communities, and 253 (24.8%) serve large communities; 67 respondents did not answer the question. Most respondents indicated that their organization engaged in at least 1 health partnership in the last 5 years, with an average of 5 partnerships.

Results indicated that as community size increased, the mean number of health partnerships increased significantly (*F* = 13.64, degrees of freedom [df] = 4, *P* < .001). Also, as community size increased, experience with partnerships increased (*F* = 9.64, df = 3, *P* < .001). Very small communities (mean, 3.8) had significantly less experience with partnerships than both medium (mean, 4.1) and large communities (mean, 4.2). Moreover, very small communities (mean, 3.8) were significantly less likely than large communities (mean, 4.1) to acknowledge the need to develop health partnerships (*F* = 3.56, df = 3, *P* = .014).

Types of health and wellness partnerships engaged in also significantly varied by community size for 7 of the 13 partner types (Table 1). Very small and large communities partnered significantly more frequently with senior services, nonprofit health promotion agencies, and public health agencies than did small and medium communities. Large and very small communities collaborated with colleges and universities, transportation agencies, and planning organizations more often than did small and medium communities. Finally, large communities most often partnered with corporations, followed by very small communities, small, and medium communities. No differences existed between community size and partnerships with hospitals, nursing homes and assisted living centers, sports organizations, schools, and health insurance companies.

**Table 1:** Chi-Square Analysis for Types of Partnerships Engaged in by Community Parks and Recreation, by Community Size^a^, 2007

Partner Type, n (%)	Very Small, n (%)	Small, n (%)	Medium, n (%)	Large, n (%)	χ^2^ (df = 3), *P* Value
Senior services	145 (33.8%)	100 (23.3%)	64 (14.9%)	120 (28.0%)	7.61, *P* = .05
Nonprofit health promotion agencies	170 (33.3%)	112 (22.0%)	77 (15.1%)	151 (29.6%)	14.57, *P* = .002
Public health agencies	204 (35.4%)	124 (21.5%)	81 (14.1%)	167 (29.0%)	16.37, *P* = .001
Colleges and universities	88 (28.6%)	59 (19.2%)	54 (17.5%)	107 (34.7%)	32.81, *P* < .001
Transportation agencies	44 (26.5%)	38 (22.9%)	22 (13.3%)	62 (37.4%)	18.82, *P* < .001
Planning organizations	47 (27.0%)	42 (24.1%)	25 (14.4%)	60 (34.5%)	14.57, *P* = .002
Corporations	44 (22.6%)	37 (19.0%)	30 (15.4%)	84 (43.1%)	47.49, *P* < .001

a Community size was defined as 1) very small (<30,000 residents), 2) small (30,000–60,000 residents), 3) medium (60,001–100,000 residents), and 4) large (≥100,001 residents).

Four of the 12 partnership effectiveness items differed significantly by community size: 1) our health partnership decision making is inclusive, 2) our health partnerships are empowered to make decisions, 3) our health partnerships have clearly defined goals and objectives, and 4) our health partnerships have a strong vision of their mission and purpose (Table 2). Regarding inclusive decision making, small and large communities reported the highest agreement followed by very small and medium communities. Regarding being empowered to make decisions and having clear goals and objectives, large communities agreed the most, followed by small, very small, and medium communities. Finally, large communities were most likely to agree that their health partnerships have a strong mission and coherent vision of their mission. Very small and small communities had the next highest ratings, and medium communities had the lowest ratings. Differences in perceived effectiveness across groups, although significant, were modest.

**Table 2:** Analysis of Variance for Mean Differences in Effectiveness of Community Parks and Recreation Partnerships, by Community Size^a^, 2007

Effectiveness Item	Very Small, Mean (SD)	Small, Mean (SD)	Medium, Mean (SD)	Large, Mean (SD)	*F* (df = 3), *P* Value
Partnership decision making is inclusive	3.4 (0.8)	3.5 (0.8)	3.3 (0.9)^b^	3.6 (0.8)^b^	2.80, *P* = .03
Partnerships are empowered to make decisions	3.5 (0.8)	3.6 (0.7)	3.4 (0.9)^b^	3.6 (0.8)^b^	3.74, *P* = .01
Partnerships have clearly defined goals and objectives	3.3 (0.8)^b^	3.4 (0.9)	3.2 (0.8)^b^	3.5 (0.9)^b^	4.18, *P* = .008
Partnerships have a strong vision of their mission statement and purpose	3.4 (0.8)	3.4 (0.8)	3.3 (0.8)^b^	3.6 (0.8)^b^	2.67, *P* = .04

a Community size was defined as 1) very small (<30,000 residents), 2) small (30,000–60,000 residents), 3) medium (60,001–100,000 residents), and 4) large (≥100,001 residents). Answers were rated on a 5-point Likert-type scale (1 = strongly disagree, 2 = disagree, 3 = neither agree nor disagree, 4 = agree, and 5 = strongly agree).

b Indicates significant group differences from the Tukey’s post-hoc tests.

Four partnership outcomes varied significantly by community size: 1) the extent to which the agency leveraged additional resources because of the partnership, 2) the ability to meet the mission statement, 3) linking to funding opportunities, and 4) making policy changes (Table 3). For all outcomes, the largest communities were more likely to report that their partnership helped them achieve these outcomes. Because outcome ratings increased as community size increased, it appears that large communities have an advantage over very small communities with regard to leveraging partnership outcomes, but these differences were modest.

**Table 3:** Analysis of Variance for Mean Differences in Community Parks and Recreation Partnership Outcomes, by Community Size^a^, 2007

Outcome	Very Small, Mean (SD)	Small, Mean (SD)	Medium, Mean (SD)	Large, Mean (SD)	*F* (df = 3), *P* Value
Extent to which the agency could leverage additional resources	3.2 (1.1)^b^	3.5 (1.1)	3.5 (1.1)	3.6 (1.1)^b^	5.62, *P* = .001
Ability to meet the mission statement	3.6 (1.1)^b^	3.7 (.8)	3.7 (1.0)	3.9 (1.0)^b^	5.06, *P* = .002
Linking to funding opportunities	3.1 (1.1)^b^	3.2 (1.0)	3.3 (1.2)	3.5 (1.0)^b^	5.12, *P* = .002
Making changes in policy	2.3 (1.1)^b^	2.6 (1.1)	2.6 (1.1)	2.7 (1.1)^b^	4.44, *P* = .004

a Community size was defined as 1) very small (<30,000 residents), 2) small (30,000–60,000 residents), 3) medium (60,001–100,000 residents), and 4) large (≥100,001 residents). Answers were rated on a 5-point Likert-type scale (1 = not at all, 3 = somewhat, and 5 = a great deal).

b Indicates significant group differences from the Tukey’s post-hoc tests.

## Discussion

As community size increased, the number of partnerships and experience with health collaborations also increased. As community size increases, the number of available partners (ie, number and type) is likely to increase as a function of population density; this is especially relevant for rural areas that comprised more than 70% of the respondents who reported working in very small communities. These findings are consistent with those of Beatty, Harris, and Barnes’ (12) partnership study, which indicated that small rural communities had significantly fewer partnerships with nonprofits than suburban and urban health departments. Only 16% all US nonprofits are in rural areas, compared with 39% in suburban and 45% in urban areas (13). Very small communities were significantly less likely to recognize the need for health partnerships. However, as suggested by others (14,15), this may be due to very limited agency budgets (because of lower tax bases), which translate to fewer staff who are spread thin in their duties. In other words, very small communities may not recognize the need to partner mainly because they are small and focused on maintaining what they have in place.

Very small and large communities were more likely than small and medium communities to partner with 7 organization types (ie, senior services, nonprofit health promotion agencies, public health agencies, colleges and universities, transportation agencies, planning organizations, and corporations). According to Gazley and Brudney (16), “The motivation to partner is driven by a desire to secure those resources most scarce for the respective sector: expertise and capacity for government, funding for nonprofits” (p. 389). This might be one reason why very small and large communities were more likely to partner with these organization types than small and medium communities. Perhaps large communities are good at recognizing when they need more expertise whereas very small communities are seeking to expand their limited capacity. Among agencies that had not engaged in partnerships during the last 5 years, the reason most frequently cited was not having enough resources to start a partnership. Resources can mean many things to people, and post-hoc analysis of open ended comments indicated that lack of staff and time and limited funding were the most common challenges to starting partnerships.

Large communities had the highest ratings of effectiveness for all 4 items (ie, inclusive decision making, empowerment to make decisions, clearly defined goals and objectives, strong vision of their mission); medium communities had the lowest ratings for 3 effectiveness items. Evidence supports the idea that clearly defined goals are particularly important to effective partnerships (17–19). For example, Gazley and Brudney (16) suggested that one reason that larger public agencies engage in partnerships is to better leverage the shared goals of the various partners. Also, it is plausible that large communities have more effective partnerships than small communities. Our data indicated that large communities were more likely to recognize the need for partnerships and they reported more experience with them. The literature suggests that having good communication at all levels, building trust, developing shared goals and values, and cooperating through coordinated actions are all important to the effectiveness of partnerships (18). Thus, it is possible that the more experience agencies or managers have with partnerships, the better able they are to meet the above effectiveness criteria.

Large communities were significantly more likely to leverage additional resources and meet their mission statement as a result of their participation in partnerships. This may be attributed to their involvement with more partnerships than smaller communities and their ability to leverage the additional resources available as a result of those partnerships. Very small communities were significantly less likely to link to funding opportunities because of their limited partnership involvement. An ongoing challenge for rural areas is that little funding is channeled to them compared with urban and suburban communities. Only 0.2% of foundation funding in the United States is awarded to small rural communities (20). Often, the smallest rural communities have organizations with few staff, sometimes only part-time, and less specialized, thereby limiting their capacity to pursue grants, sponsorships, and other funding opportunities (12). Also, according to Mays and Scutchfield (21), smaller communities often have few organizations in their communities to address health needs compared with larger communities that have more decentralized governance, which results in partnerships that are more entrepreneurial. The literature on barriers that limit partnerships supports the assertion that smaller agencies might experience more challenges with partnership development and management than larger ones. For example, barriers reported by respondents in a study by Brownson et al (22) were bureaucracy, interorganizational policies, lack of time, differing organizational goals, challenges finding collaborators, and the costs of partnering outweighing the benefits. Moreover, Kegler et al (23) suggested that turf issues and competition were also issues of concern regarding limited resources and budget concerns.

The differences in effectiveness and outcomes related to partnerships might also be about leadership. According to Bryson et al (24), “the leadership challenge in cross-sector collaboration may be viewed as a challenge of aligning initial conditions, processes, structures, governance, contingencies and constraints, outcomes, and accountabilities such that good things happen in a sustained way over time — indeed, so that public value can be created” (p. 52). In our sample, respondents who work in large communities had the most years of work experience in their agency. It is likely that directors with more experience are better equipped to face the challenges presented when creating and managing partnerships. Kegler et al (23) suggested that it takes a special type of leader to successfully create and manage partnerships: a boundary leader. They asserted that boundary leaders are people who can skillfully negotiate the spaces between organizations. Moreover, Tower et al (17) posited that a feature of successful partner relationships is complementary expertise and knowledge among the players in the partnership.

One of the limitations of this study is that we did not explicitly define the concept of “partnership” for respondents. Therefore, various partnership types (ie, short, long-term, formal, and informal) were likely reported on. Nevertheless, Cousens et al (25) reported that “regardless of the type or pattern of linkage, participants identified their relationships as a partnership” (p. 32) and that it appeared to be a “broadly encompassing phrase used to indicate an external linkage” (p. 42). As a next step, it would be useful to better understand the essential elements of different types of partnerships between community parks and recreation and health organizations. For example, does effectiveness vary depending on how long the partnership has existed, the complexity and number of organizations involved in the partnership, or by the sectors involved in the partnership? These questions are important to answer as community park and recreation agencies continue to engage in health partnerships. Our findings describe some differences that exist in partnerships by community size; however, the mechanisms that facilitate perceptions of partnership effectiveness and outcomes remain unclear. Partnership practices should be examined to better understand the processes that shape the effectiveness and outcomes of these health partnerships.

Despite these limitations, some differences regarding partnership practices, effectiveness, and outcomes were revealed among different sizes of communities. These findings indicate that smaller communities (which are most likely rural) are disadvantaged regarding their ability to develop and implement partnerships with health organizations. This is especially troubling since poverty, obesity, chronic disease, and uninsured rates are higher in rural areas than suburban and urban areas (26,27). Not only do very small communities engage in partnership less, they have fewer organizations to partner with and they must serve a significantly larger geographic area (49.1 miles for rural vs 0.5 miles in urban areas) (28). Moreover, their partnerships were less effective than those of larger communities and they were less able to leverage additional resources and funding from their partnerships. Thus, there is a need to focus on increasing the partnership education and training efforts for park and recreation agencies in small towns and rural communities. Simultaneously, rural advocacy organizations could work with foundations and government agencies to raise awareness of the critical need for resources that will facilitate effective partnerships between community parks and recreation and health organizations. These efforts should improve agencies’ abilities to develop and maintain partnerships that more effectively impact public health goals.
